# Supplementary material for: MetaRibo-Seq measures translation in microbiomes
Source: Nat Commun. 2020 Jun 29;11:3268. doi: 10.1038/s41467-020-17081-z (PMC7324362; doi:10.1038/s41467-020-17081-z)
Supplement: Supplementary file 10 — Supplementary Data 7 [file 41467_2020_17081_MOESM10_ESM.zip › File2/Confidence_High_Taxonomy/69681_out.krona.html]

Javascript must be enabled to view this page.

members
magnitude
magnitudeUnassigned
count
unassigned
taxon
rank

69681\_out

2

SRS893252\_contig\_number\_contig-100\_2434.95241
1

superkingdom
1
2

1239
1
phylum

class
526524
1

order
526525
1

family
1
128827

genus
1573535
1

species
1735

SRS053356\_contig\_number\_contig-100\_608.268123
1
